# B7-H3 and CSPG4-targeted CAR T cells as potent effectors in anaplastic thyroid cancer

**DOI:** 10.1186/s13046-025-03475-8

**Published:** 2025-08-22

**Authors:** Giulia Cattaneo, Marco Ventin, Shahrzad Arya, Cedric Bailey, Venkata Rao Vantaku, Jingyu Jia, Maoyang Qi, Luke Maggs, Xinhui Wang, Sareh Parangi, Soldano Ferrone, Cristina R. Ferrone

**Affiliations:** 1 Division of Gastrointestinal and Surgical Oncology, Department of Surgery, Massachusetts General Hospital, Harvard Medical School, Boston, MA USA; 2 Department of Surgery, Cedars-Sinai Medical Center, Los Angeles, CA USA; 3 Department of Pathology & Laboratory Medicine, Cedars-Sinai Medical Center, Los Angeles, CA USA; 4 Department of Surgery, Massachusetts General Hospital, Harvard Medical School, Boston, MA USA

**Keywords:** Anaplastic thyroid cancer, Immunotherapy, CAR T, CSPG4, B7-H3

## Abstract

**Background:**

Anaplastic thyroid cancer (ATC) is a rare and aggressive malignancy with poor survival and no available effective therapy. This unmet clinical need led us to investigate chimeric antigen receptor (CAR) T cells s as potential treatment option for this malignant disease. As target tumor antigens of our CAR T cell therapy, we selected the chondroitin sulfate proteoglycan 4 (CSPG4) and the B7-homolog 3 (B7-H3), as they are both highly and homogeneously expressed on different types of thyroid carcinoma cell lines and tissues, including ATC. Importantly, both CSPG4 and B7-H3 have a low distribution on normal tissues, thus limiting ‘on-target off-tumor’ CAR T-related toxicities.

**Methods:**

We generated CSPG4-specific and B7-H3-specific CAR T cells by utilizing a second-generation CAR construct comprised of a CD28 costimulatory domain and tested their antitumor activity in vitro and in an orthotopic xenograft murine model of ATC.

**Results:**

We demonstrated that thyroid cancer cells are specifically recognized and effectively eradicated in vitro by CSPG4-targeted and B7-H3-targeted CAR T cells. Additionally, both CAR T cell types were able to mediate significant control or complete eradication of primary ATC tumors when mice were treated with CSPG4 CAR T cells or B7-H3 CAR T cells, respectively.

**Conclusion:**

Overall, in this study we identified CSPG4 and B7-H3 as valuable target antigens in thyroid cancer and demonstrated that CAR T cell immunotherapy can be a valuable therapeutic option for ATC patients. Our findings provide the translational basis for exploring CAR T cell immunotherapies targeting CSPG4 and B7-H3 with ATC patients who do not respond or relapse after first line treatment.

**Supplementary Information:**

The online version contains supplementary material available at 10.1186/s13046-025-03475-8.

## Background

The clinical outcome of thyroid cancer patients is strictly related to the tumor pathological subtype. Differentiated thyroid cancers, which can be classified as papillary thyroid carcinoma (PTC), and follicular thyroid carcinoma (FTC) represent most cases and respond well to standard treatments [1]. In contrast, patients with poorly differentiated tumors have a worse clinical outcome. In particular, anaplastic thyroid cancer (ATC) has the highest mortality rate (close to 100%) among all thyroid cancer subtypes [2]. Local disease progression and distant metastases rapidly occur, and most ATC patients inevitably succumb to their disease, with a median survival rate of 6 months [3].

Currently available therapies for ATC are not effective or provide only limited clinical benefits [2]. Patients with advanced disease harboring BRAF-mutated tumors can benefit from the FDA-approved combination of dabrafenib (BRAF inhibitor) and trametinib (MEK inhibitor) [4]. However, most patients do not achieve a complete response and experience disease progression because of the development of therapeutic resistance.

To address this unmet clinical need, we investigated the therapeutic potential of an immunotherapeutic strategy based on the use of T cells specifically redirected with a chimeric antigen receptor (CAR). CARs are recombinant proteins comprised of an extracellular domain in most cases derived from the single chain variable fragment (scFv) of a monoclonal antibody (mAb), which mediates the specific binding with a target tumor antigen (TA) selectively expressed on cancer cells, and an intracellular portion which includes the CD3ζ signaling domain and one or more co-stimulatory domains [5].

As target TAs of our CAR T cell-based therapy we selected the chondroitin sulfate proteoglycan 4 (CSPG4) and the B7 homolog 3 (B7-H3). Both TAs have been shown to be functionally involved in tumor growth, disease progression, and metastatic spread in different types of solid tumors [6–8] and overexpression of either antigen is a poor prognostic factor in solid cancer patients, including ATC [9, 10]. Due to their homogeneous distribution across several solid tumors, CSPG4 and B7-H3 represent promising and attractive targets for CAR-redirected T cells, and their limited distribution in normal tissues increases the safety profile of this immunotherapeutic strategy [6, 11]. In our previous work we have reported the strong antitumor activity of B7-H3 CAR T cells and CSPG4-CAR T cells against different types of solid cancers in vitro and in murine models as monotherapy as well as in combination with radiation therapy [12–14].

In this study, we demonstrate the preclinical therapeutic potential of targeting CSPG4 and B7-H3 with CAR T cells for the treatment of anaplastic thyroid cancer.

## Methods

### Cell lines

The human ATC cell line 8505c (BRAF, NF2, TERT and TP53 mutated) and the human PTC cell line BCPAP (BRAF, TERT and TP53 mutated) were purchased from DSMZ. The human ATC cell line SW1736 (BRAF, TERT, TP53 and TSH3 mutated) was kindly provided by Dr. S. Parangi (Massachusetts General Hospital).The human 2 C-HCC hurthle cell carcinoma cell line was generated from a patient-derived xenograft murine model. The 8505c cell line was transduced with a lentiviral vector encoding green fluorescent protein (GFP) and luciferase (Luc) and referred to as 8505c-GFP.Luc. Viral supernatant was produced by transfection of HEK293T cells (purchased from ATCC) with the pMD2.G plasmid (envelope), the psPAX2 plasmid (gag-pol), and the the GFP.Luc plasmid (kindly provided by Dr. J Franses, Massachusetts General Hospital). Thyroid cancer cell lines were cultured in Dulbecco Modified Eagle Medium (DMEM, Sigma-Aldrich, Cat# D6429) supplemented with 10% fetal bovine serum (FBS) (GeminiBio, Cat# S11150), in a humidified 5% CO2 incubator at 37 °C. The human pancreatic cancer cell line PDAC6, previously generated by Dr. CR. Ferrone from the ascites of a pancreatic cancer patient, was cultured in DMEM supplemented with 10% FBS [15]. The Jurkat and MDA-MB-231 cell lines were purchased from ATCC and cultured in RPMI-1640 (Corning^®^, Cat# 10-043-CVR) supplemented with 10% FBS.

### Immunohistochemistry

The tissue microarray (TMA) slide was purchased from TissueArray (ID #TH208a) and treated according to the IHC staining procedure which has been set up and optimized by the Pathology Core at the Massachusetts General Hospital. As a first step, the slide was baked overnight to ensure optimal adhesion of the tissue to the slide. Then, the slide was warmed up to 91 °C and treated with CC2 retrieval solution (NaCit) (Ventana, Cat# 950 − 223). The primary CSPG4-specific mouse anti-human 763.74 mAb, produced by Dr. S. Ferrone from the 763.74 hybridoma cell line, was utilized at the concentration of 4 µg/ml and incubated for 40 min at 37 °C. Anti-mouse HRP (Ventana, Cat# 760–4311) was utilized asdetection method and incubated for 60 min with the primary antibody. The reaction was visualized with DAB chromogen substrate (ChromoMap DAB Kit (RUO), Roche, Cat# 760 − 159) and bluing reagent (Ventana, Cat# 760–2037), which were applied as counterstain for 60 min. Cover slips were added using histological mounting medium (Fisher Scientific, Cat# SP15-100). The slide was imaged with a digital slide scanner (Pannoramic 250, 3D Histech, Hungary) and visualized using the NDP.view2 Viewing software. CSPG4 expression was evaluated by a board-certified attending pathologist, by using a standard scoring system. Specifically, the score indicating the percentage of stained cells was defined as 0, 1, 2 or 3 when the percentage of positive cells was 0, 1–29, 30–59 or 60–100, respectively. The score indicating the intensity of staining was defined as 0, 1, 2 or 3 when the intensity was negative, weak, intermediate or strong, respectively [10]. The overall score was calculated by adding the score of percentage and the score of intensity together to generate a scaled score of CSPG4 expression, defined as follows: negative (0), weak (1–2), intermediate (3–4) or strong (5–6) [10].

### CAR T cell generation

The B7-H3-specific CAR construct was generated by using the scFv derived from the 376.96 mouse mAb, an IgG2 which specifically recognizes a B7-H3 epitope, while the CSPG4-specific CAR construct was generated by using the scFv derived from the 763.74 mouse mAb, an IgG1 which specifically recognizes a CSPG4 epitope [16–18]. CAR T cells were generated as previously described [16]. Briefly, peripheral blood mononuclear cells (PBMCs) were isolated by density gradient centrifugation (Lymphoprep™, STEMCELL, Cat# 18061) of buffy coats obtained from normal donors (Research Blood Components) and plated in a 24-well non-treated tissue culture plate (ThermoFisher Scientific, Cat# 144530) pre-coated with anti-CD3 Ab (Miltenyi Biotec, clone OKT3, Cat# 130-093-387) at the concentration of 1 µg/ml and anti-CD28 Ab at the concentration of 1 µg/ml (BD Bioscience, Cat# 556620), to induce T cell activation. IL-7 (PeproTech^®^, Cat# 200-07) and IL-15 (PeproTech^®^, Cat# 200 − 15) were added to the culture medium at the concentration of 10ng/ml and 5ng/ml, respectively. Retroviral supernatant was added to retronectin-coated tissue culture plates (Takara Bio, Cat# T100A) and centrifuged at 2000 g for 90 min. After removal of the viral supernatant, activated T cells were plated at the concentration of 4 × 10^5^ cells per well, and centrifuged at 1000 g for 10 min. Following a 48 h incubation, fresh culture medium supplemented with IL-7 andIL-15 was added to allow CAR T cell expansion. Retroviral supernatants utilized for the transduction of human T cells were produced by transfection of HEK293T cells with the RDF plasmid (envelope), the PegPam3 plasmid (gag-pol), and the retroviral vector encoding the CSPG4-specific CAR, B7-H3-specific CAR or CD19-specific CAR kindly provided by Dr. Gianpietro Dotti (University of North Carolina at Chapel Hill, Chapel Hill, NC, USA).

### Coculture experiments

***3-(4***,***5-dimethylthiazol-2-yl)-2***,***5-diphenyl-2 H-tetrazolium bromide (MTT) assay.*** Cancer cells were seeded in 96-well tissue culture plates at the concentration of 5000 cells perwell. CAR T cells were added to the plate at multiple effector (CAR T) to target (cancer) ratios without the addition of exogenous cytokines, and cultured for 72 h. At the end of the incubation, cancer cell viability was assessed by MTT assay (Millipore Sigma, Cat# M5655) according to manufacturer’s instructions.

***Live cell-imaging.*** 8505c cells were plated in a 96-well tissue culture plate at the concentration of 5000 cells per well. CAR T cells were labeled with the PKH26 Red Fluorescent Cell Membrane Dye (Millipore Sigma, Cat# PKH26GL) according to manufacturer’s instructions and added to the cell culture without the addition of exogenous cytokines. Tumor cell proliferation was monitored using an Incucyte^®^ SX5 Live-Cell Analysis Instrument over a 5-day incubation.

***Flow cytometry.*** Jurkat cells were labeled with the PKH26 Red Fluorescent Cell Membrane Dye according to manufacturer’s instructions and plated in 96-well tissue culture plate at the concentration of 5000 cells per well. CAR T cells were added at the indicated effector to target ratios and incubated for 72 h. At the end of the incubation, cells were collected and the number of residual Jurkat cells was determined by flow cytometry by utilizing propidium iodide (PI) as viability marker.

### Effector cytokines release assay

Target cells cells were seeded in 24-well tissue culture plates at the concentration of 50.000 cells per well. CAR T cells were added to the cell culture at the 1:1 effector to target ratio without the addition of exogenous cytokines. Following a 24-hour incubation period, cell culture supernatants were collected, and T cell effector cytokines were measured utilizing a bead-based immunoassay (LEGENDplex™ V-Bottom Multiplex Assay kit for Human CD8/NK Panel, Cat# 741065) according to manufacturer’s instructions.

### Flow cytometry

CSPG4 and B7-H3 expression on thyroid cancer cells was assessed by using the CSPG4-specific 763.74 and the B7-H3-specific 376.96 mouse anti-human mAbs at the concentration of 1µg/ml. Following a 45-minute incubation at 4°C with the primary antibodies, cells were washed with PBS containing 0.5% BSA and stained with a FITC-conjugated goat anti-mouse secondary antibody (Jackson ImmunoResearch, Cat# 115-095-003) (1:100 dilution) for detection. B7-H3 CAR T cell transduction efficiency was assessed by utilizing a FITC-Labeled Human B7-H3 protein (4Ig) (Acro Biosystem, Cat# B7B-HF2E7) at the concentration of 2µg/ml, or by using an AlexaFluor 647-conjugated F(ab’) ₂ fragment goat anti-mouse IgG F(ab’)₂ - specific (Jackson ImmunoResearch, Cat# 115-606-072); CSPG4 CAR T cell transduction efficiency was assessed by utilizing the MK2-23 anti-idiotype at the concentration of 1 µg/ml. Mouse anti-human PacificBlue-conjugated anti-CD69 mAb (Biolegend, Cat# 310919, 1:100 dilution) was utilized to measure CD69 expression on CAR T cells. Mouse anti-human PE-Cy7-conjugated anti-CD3 mAb (Biolegend, Cat# 344815, 1:100 dilution) and PE-conjugated anti-CD45 (Biolegend, Cat# 368509, 1:100 diluition) were utilized to detect the presence of T cells in the blood of mice and CountBright™ Absolute Counting Beads (ThermoFisher Scientific, Cat# C36950) were added to calculate the number of circulating T cells. Samples were acquired with Cytek^®^ Aurora Flow Cytometry System and results were analyzed utilizing FlowJo^™^ 10.9 software.

### Orthotopic xenograft murine model of ATC

All mouse experiments were performed in accordance with Institutional Animal Care and Use Committee (IACUC) guidelines.

***Mice.*** Female NOD-*scid* IL2Rgamma^null^ (NSG) 8–10 weeks old mice were purchased from the Jackson laboratory (Strain #: 005557) and housed in the Animal Core Facility at the Massachusetts General Hospital.

***Orthotopic model.*** The ATC orthotopic murine model was generated as previously described [19]. Briefly, a horizontal incision was performed to expose the central part of the neck. Salivary glands were reflected laterally, and overlaying muscles were dissected away from the right thyroid. The 8505c-GFP.Luc cell line (500.000 cells/mouse resuspended in 5ul of PBS) was injected into the right thyroid by utilizing a Hamilton syringe. Following the injection, the needle was slowly retracted, and the salivary gland were relocated to their original position. The incision was closed with continuous suture and antibiotic cream was applied to prevent wound infection.

### Bioluminescence imaging

Tumor growth was monitored by Bioluminescence Imaging (BLI). Mice were anesthetized using 2% isoflurane in oxygen and intraperitoneally injected with D-luciferin Firefly. Fluorescence intensity was measured by AMI HTX in vivo imaging system (Spectral Instruments Imaging). Image processing and the quantification of photon flux was performed with Aura Imaging software.

### Statistical analysis

Data were analyzed by using GraphPad Prism 9 software (VERSION 9.5.1) and presented as mean values ± SD of three independent experiments. One-way ANOVA with Tukey’s multiple comparison test was utilized to determine statistical significance in the comparison among groups. Kaplan-Meier survival curves were analyzed by log-rank test. Significance is represented in graphs as ns for not significant, * for *p* ≤ 0.05, *** for *p* ≤ 0.001, **** for *p* ≤ 0.0001. CSPG4 and B7-H3 mRNA expression levels were obtained from a publicly available database (accession number GSE126698) which includes RNA sequencing data obtained from primary thyroid tumors (*n* = 22), including ATC, FTC and PTC, and normal thyroid tissues (*n* = 6). RNA expression data were analyzed by utilizing the R package GEOquery.

## Results

### CSPG4 is highly and homogeneously expressed across different types of thyroid cancers

**Fig. 1:**
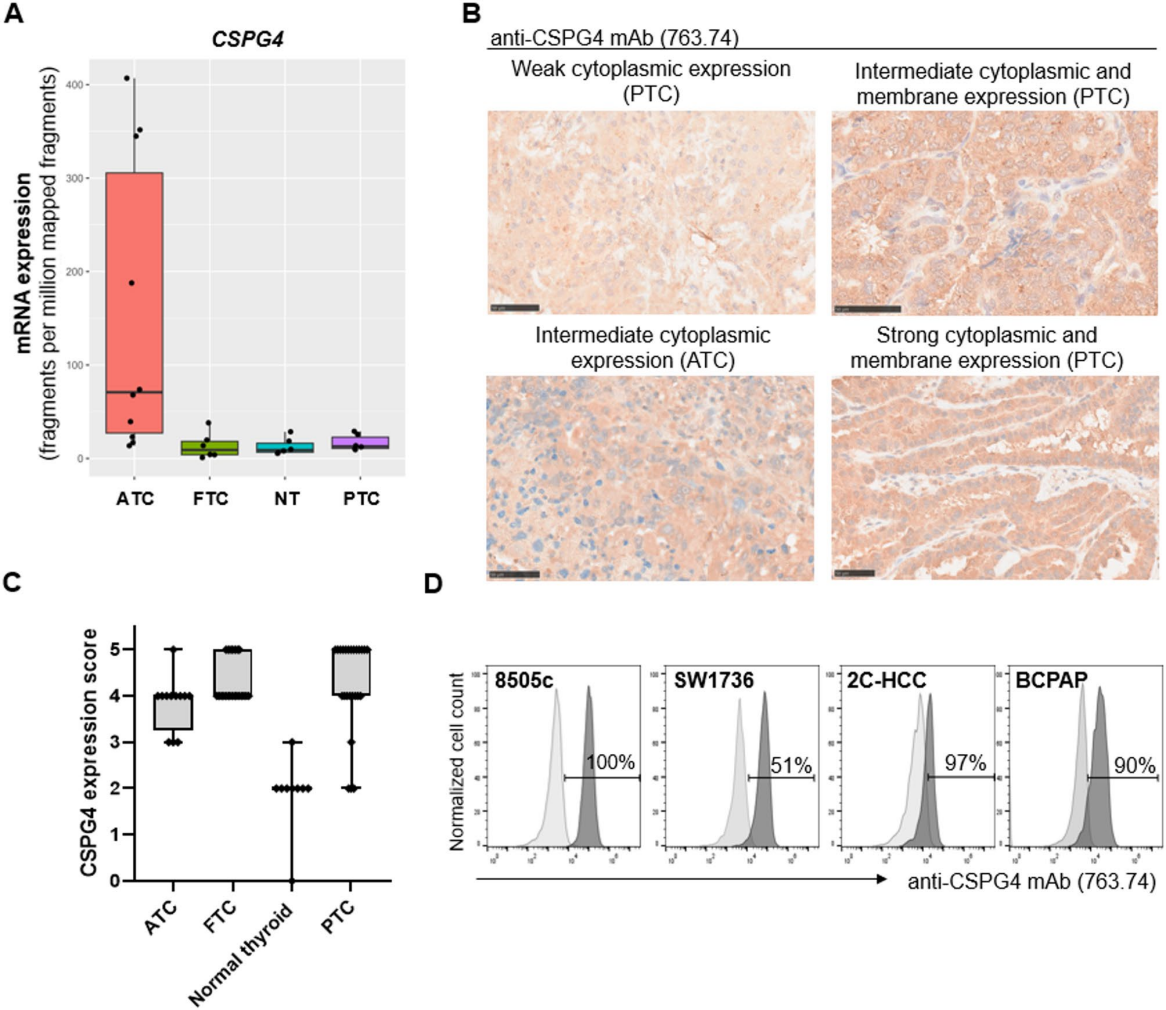
CSPG4 is highly and homogeneously expressed in thyroid cancers. **(A)** Box plot showing *CSPG4* mRNA expression level in patients with anaplastic thyroid cancer (ATC), follicular thyroid carcinoma (FTC), papillary thyroid carcinoma (PTC), and in normal thyroid tissues (NT). Data were analyzed by utilizing a publicly available database (accession number GSE126698). **(B)** Representative images of primary thyroid tumors demonstrating high expression of CSPG4, assessed by IHC staining performed with the CSPG4-specific mAb 763.74 (4 µg/mL) (400x, scale bar 50 μm). **(C)** Box plot summarizing the overall score of CSPG4 expression on primary thyroid tumors, assessed by quantifying the percentage of positive stained cells and the intensity of expression. **(D)** Membrane expression of CSPG4 on human thyroid cancer cell lines assessed by flow cytometry by utilizing the CSPG4-specific 763.74 mAb; the MK2.23 mAb was utilized as isotype control

To investigate the potential clinical relevance of targeting CSPG4, we first evaluated its expression profile across thyroid cancers. We started by assessing *CSPG4* mRNA expression level by utilizing a publicly available database (accession number GSE126698) [20] which includes human samples of anaplastic thyroid cancer (ATC), follicular thyroid carcinoma (FTC), papillary thyroid carcinoma (PTC), and normal thyroid tissues as control. ATC samples demonstrated a higher CSPG4 expression, with some variability across patients, compared to FTC and PTC samples, in which the expression level was similar to the one observed in normal thyroid tissues (Fig. 1A). To determine whether high mRNA levels correlate with protein expression, we evaluated the presence of CSPG4 on human tissue samples of thyroid cancers. CSPG4 expression was evaluated using an immunoreactivity score assessment. The analyzed cancer samples showed CSPG4 expression that was either strong (PTC = 11/25, FTC = 7/22, ATC = 1/12), intermediate (PTC = 11/25, FTC = 15/22, ATC = 11/12), or weak (PTC = 3), with patterns of expression including cytoplasmic (43 of 59 cancer samples) and combined membranous and cytoplasmic (16 of 59 cancer samples); contrarily, the nine non-cancer thyroid samples demonstrated low or negative CSPG4 expression (Fig. 1B, C, Supplementary Fig. 1A, B). Patient’s demographic information and detailed score assessment is described in Supplementary Table 1.

### CAR T cells specifically recognize and effectively eliminate thyroid cancer cells in vitro

To investigate the in vitro antitumor activity of CSPG4-targeted CAR T cells, we first assessed CSPG4 membrane expression on 4 human thyroid cancer cell lines, including 8505c and SW1736 (ATC), 2 C-HCC (hurthle cell carcinoma) and BCPAP (PTC). We found that CSPG4 was highly expressed on all the analyzed cell lines, with a mean of 84 ± 22% of positive stained cells (Fig. 1D).

**Fig. 2:**
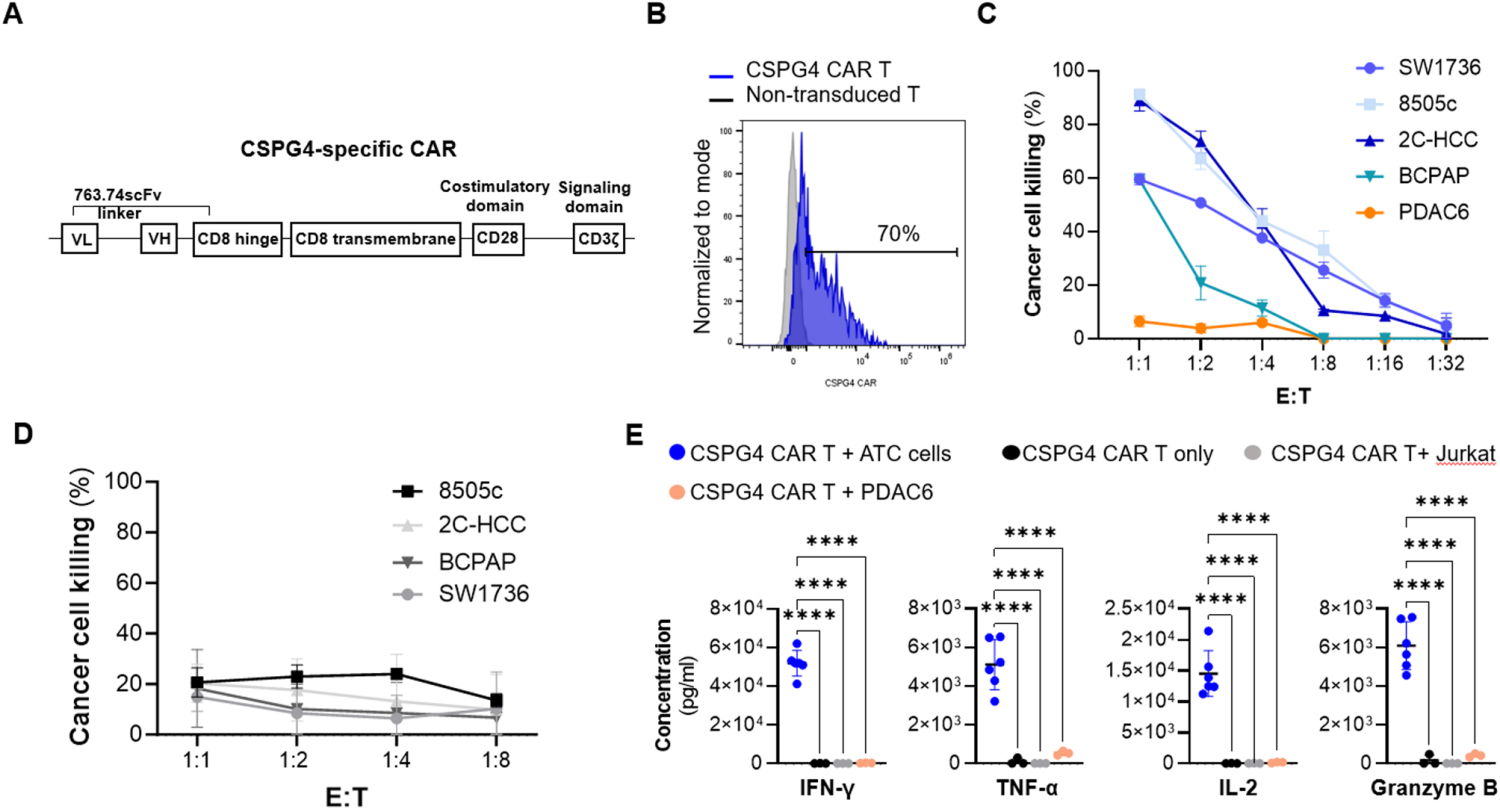
CSPG4 CAR T cells specifically recognize and effective eradicate thyroid cancer cells in vitro. **A)** Schematic representation of the CSPG4-specific CAR construct. **B)** Representative flow cytometric histogram demonstrating CSPG4 CAR T cell transduction efficiency. **C)** Cumulative curves demonstrating the percentage of cancer cell killing mediated by CSPG4 CAR T cells against 4 thyroid cancer cell lines and the pancreatic cancer cell line PDAC6 after a 72-hour coculture period. **D)** CD19 CAR T cell-mediated antitumor activity against thyroid cancer cell lines. **E**) Levels of effector cytokines released in the cell culture supernatant by CAR T cells after a 24-hour coculture with 8505c and SW1736 cells (CSPG4 positive) or Jurkat and PDAC6 cells (CSPG4 negative). Results are shown as mean ± SD of *n* = 3 independent experiments. Statistical analysis was performed by one-way ANOVA multiple comparison test. ***p-value ≤ 0.001, ****p-value ≤ 0.0001

We successfully generated CSPG4 CAR T cells from three normal donors, with a mean transduction efficiency of 65 ± 10% (Fig. 2A, B). Next, we tested the in vitro antitumor activity of CSPG4 CAR T cells by co-culturing them with thyroid cancer cells at multiple effector (CAR T) to target (cancer cells) (E: T) ratios, ranging from 1:1 to 1:32. CSPG4 CAR T cells demonstrated strong antitumor activity against all the tested cell lines, by achieving near complete eradication of target cells at high ratios (cancer cell lysis of 72.2 ± 15.6% at the 1:1 E: T and 50.3 ± 20.7% at the 1:2 E: T) (Fig. 2C). We observed that the percentage of cancer cell killing was lower when CSPG4 CAR T cells were cocultured with the SW1736 and BCPAP cell lines, as compared to the 8505c and 2C-HCC cell lines. A potential explanation for this difference might be related to the level of CSPG4 expression, which was found to be lower on SW1736 and BCPAP cells (51% of positive cells with an MFI of 10764 and 90% of positive cells with an MFI of 68170, respectively), as compared to 8505c and 2C-HCC cells (100% of positive cells with an MFI of 103244 and 97% of positive cells with an MFI of 90264, respectively). This hypothesis was confirmed by the positive trend observed between target antigen expression and CAR T cell-mediated killing (Supplementary Fig. 1C). To prove the lack of CAR T cell-associated ‘off-tumor’ toxicity CSPG4 CAR T cells were cocultured with Jurkat cells and the pancreatic cell line PDAC6, which do not express CSPG4 (Supplementary Fig. 1E). As demonstrated in Fig. 2C and in Supplementary Fig. 1F and G, no killing was observed in these experimental conditions. Moreover, CAR T cells cocultured with Jurkat cells did not show signs of activation, as indicated by lack of CD69 upregulation, as compared to CAR T cells cocultured with CSPG4-expressing 8505c cells (Supplementary Fig. 1H). The specificity of CAR-mediated recognition and elimination of cancer cells was confirmed by the lack of killing observed when thyroid cancer cell lines were cocultured with CD19 CAR T cells. The latter were selected as control of the CAR specificity because CD19 is not expressed on thyroid cancers (Fig. 2D). Finally, CAR engagement-specificity was further confirmed by the significantly higher release of T cell effector cytokines (IL-2, TNF-α, IFN-γ) and Granzyme B mediated by CSPG4 CAR T cells when co-cultured with ATC cells (8505c, SW1736), as compared to CSPG4 CAR T cells cocultured with the CSPG4 negative Jurkat and PDAC6 cell lines (Fig. 2E).

### CSPG4 CAR T cells effectively control tumor growth and prevent the development of lung metastases in mice orthotopically grafted with ATC tumors

**Fig. 3:**
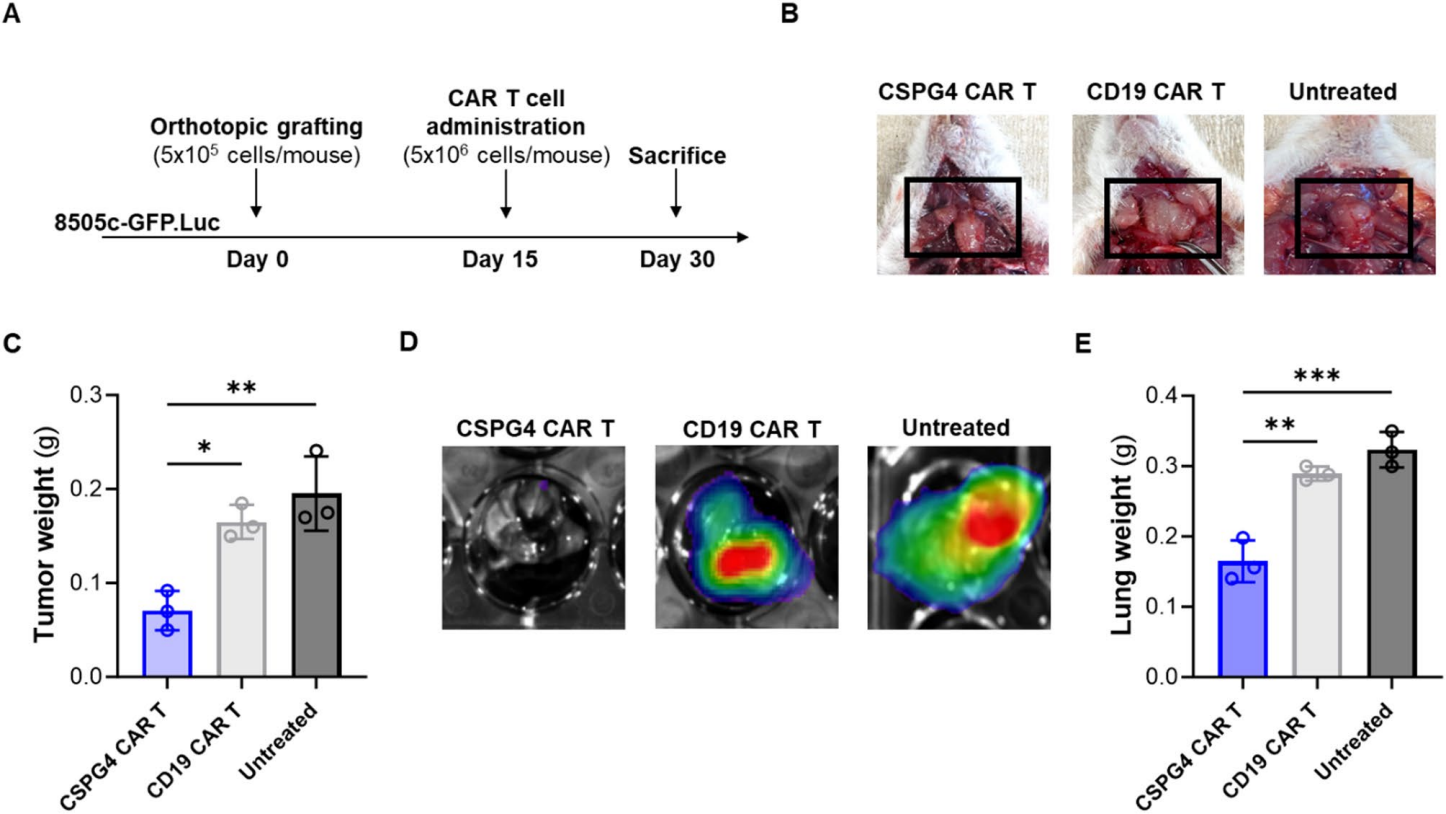
CSPG4 CAR T cells effectively control ATC tumor growth and prevent the development of lung metastases in mice. **(A)** Schematic timeline of the in vivo experiment. **(B)** Representative images of mice sacrificed 15 days after CAR T cell administration and macroscopically examined for the presence of ATC tumors (*n* = 3 mice/group). **(C)** Weight of ATC tumors collected from mice of each treatment group at the time of sacrifice. **(D)** Representative BLI images of lungs collected from mice of each treatment group at the time of sacrifice. **(E)** Weight of lungs collected from mice of each treatment group at the time of sacrifice. Statistical analysis was performed by one-way ANOVA multiple comparison test. *p-value ≤ 0.05, **p ≤ 0.01, ***p-value ≤ 0.001

To confirm in vivo the antitumor activity of CSPG4 CAR T cells, we tested their ability to eradicate thyroid tumors in a xenograft murine model of ATC. The 8505c-GFP.Luc ATC cell line was orthotopically grafted in the right thyroid of NSG mice. Fifteen days following cancer cell grafting the presence of tumors was confirmed by bioluminescence imaging (BLI), and mice were treated with CSPG4 CAR T cells or CD19 CAR T cells, administered as a single dose by tail vein injection (Fig. 3A). Fifteen days after CAR T cell administration, mice were sacrificed and examined for the presence of tumors. We observed that CSPG4 CAR T cells were able to significantly control tumor growth compared to control groups, as demonstrated by macroscopical observation, as well as by a significant reduction of tumor weight (Fig. 3B, C).

Additionally, since lungs are the most common metastatic site in ATC patients, we analyzed lungs collected from mice for the presence of cancer cells. As demonstrated by BLI images in Figs. 3D and 8505c-GFP.Luc cells were not detected in the lungs of mice treated with CSPG4 CAR T cells, as opposed to control groups. This finding was also reflected in a significant lower weight of lungs collected from the CSPG4 CAR T cell group compared to controls (Fig. 3E).

Overall, our results indicate that CSPG4 CAR T cell administration has the potential to control the growth of primary ATC tumors, as well as prevent the development of metastatic spread to the lungs. However, considering that CSPG4 CAR T cells were not able to achieve complete tumor eradication, we sought to evaluate the efficacy of CAR T cells targeting a different tumor antigen. In this regard, B7 homolog 3 (B7-H3) represents a valuable alternative target since it has been found to be highly expressed in ATC tumors [9].

### B7-H3-targeted CAR T cells are effective in eradicating thyroid cancer cells in vitro

**Fig. 4:**
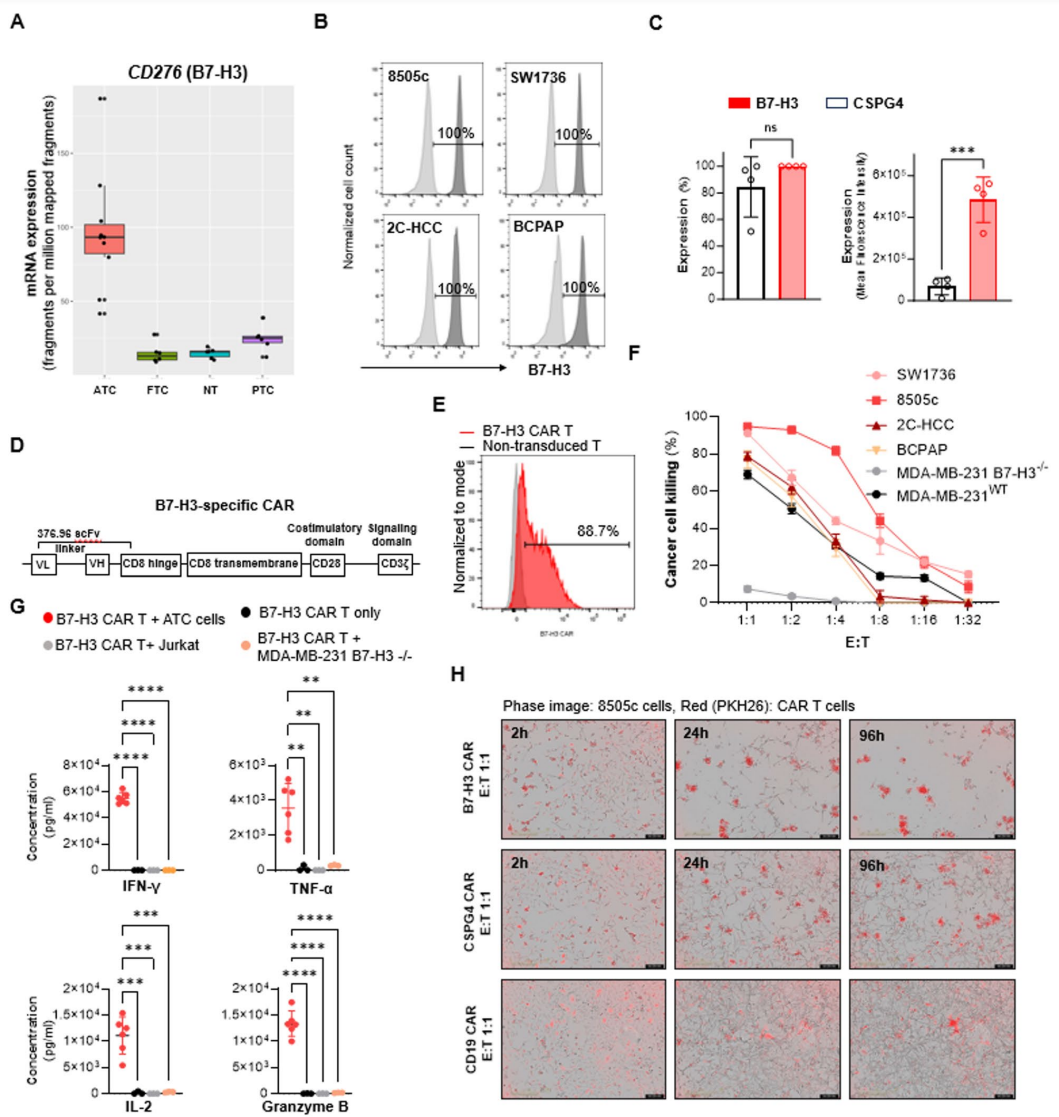
B7-H3-targeted CAR T cells effectively eradicate thyroid cancer cells in vitro. **(A)** Box plot showing *CD276* (B7-H3) mRNA expression level in patients with ATC, FTC, PTC, and in normal thyroid tissues (NT). Data were analyzed by utilizing a publicly available database (accession number GSE126698). **(B)** and **(C)** Percentage and intensity of membrane B7-H3 expression on human thyroid cancer cell lines assessed by flow cytometry. B7-H3 expression was determined by utilizing the B7-H3-specific 376.96 mAb; the F3C.25 mAb was utilized as isotype control. **(D)** Schematic representation of the B7-H3-specific CAR construct. **(E)** Representative flow cytometric histogram showing B7-H3 CAR T cell transduction efficiency. **(F)** Cumulative curves demonstrating the percentage of cancer cell killing mediated by B7-H3 CAR T cells against 4 thyroid cancer cell lines and the MDA-MB-231 B7-H3^-/-^ or MDA-MB-231 B7-H3^wt^ cell line after a 72-hour coculture period. **(G)** Levels of effector cytokines released in the cell culture supernatant by CAR T cells after a 24-hour coculture with 8505c and SW1736 cells (B7-H3 positive) or Jurkat and MDA-MB-231 B7-H3^-/-^ cells (B7-H3 negative). **(H)** Live monitoring of 8505c cell growth (phase image) cocultured with either B7-H3-specific, CSPG4-specific, or CD19-specific CAR T cells (PKH26-labeled, red) for a 96 h-coculture period at the 1:1 E: T ratio. Results are shown as mean ± SD of *n* = 3 independent experiments. Statistical analysis was performed by unpaired t-test or one-way ANOVA multiple comparison test. *p-value ≤ 0.05, **p-value ≤ 0.01, ***p-value ≤ 0.001, ****p-value ≤ 0.0001

To confirm the hypothesis that B7-H3 may represent a valuable target antigen for CAR T cell-based immunotherapy, we first evaluated its expression profile across thyroid cancers by utilizing the previously mentioned publicly available database (GSE126698). As we observed for CSPG4, B7-H3 (*CD276*) mRNA expression level was significantly higher in ATC samples, as compared to FTC, PTC, as well as normal thyroid tissues (Fig. 4A). By utilizing the previously described thyroid cancer cell lines, we found that B7-H3 had a more homogenous and intense expression than CSPG4 (100% and 84 ± 22% of positive stained cells with a mean fluorescence intensity of 68106 and 483930 for B7-H3 and CSPG4, respectively) (Fig. 4B, C).

Based on our findings, we genetically engineered normal donor T cells (*n* = 3) with a B7-H3-specific CAR construct; mean transduction efficiency was confirmed by utilizing two different detection methods (Supplementary Fig. 2) (33), and it was comparable to CSPG4 CAR T cells (70 ± 4% (Fig. 2D, E). B7-H3 CAR T cells demonstrated intense in vitro antitumor activity when cocultured with thyroid cancer cell lines at high ratios (cancer cell lysis: 87.5 ± 7.2% at the 1:1 E: T, 72.4 ± 14.4% at the 1:2 E: T), and CAR engagement-specificity was confirmed by the release of T cell effector cytokines mediated by B7-H3 CAR T cells when co-cultured with B7-H3 positive ATC cells (Fig. 4H). As observed for CSPG4 CAR T cells, B7-H3 CAR T cells did not show ‘off-tumor’ effect, as demonstrated by the lack of killing when cocultured with the B7-H3 negative Jurkat cell line or with the breast cancer cell line MDA-MB-231 genetically modified to knockout B7-H3 expression (MDA-MB-231 B7-H3^-/-^) (Fig. 2F, Supplementary Fig. 1E-I).

Overall, we observed that B7-H3 CAR T-mediated killing was higher compared to CSPG4 CAR T cells, indicating that B7-H3 may represent a more effective target antigen. These findings were confirmed by monitoring the growth of the 8505c cells over a 5-day co-culture in the presence of either B7-H3-specific or CSPG4-specific CAR T cells at the 1:1 ratio. The extent of killing was already appreciable after 24 h of co-culture, with nearly complete eradication occurring at the end of the incubation period when 8505c cells were cocultured with B7-H4 CAR T cells but not with CSPG4 CAR T cells (Fig. 4G).

### B7-H3 CAR T cells demonstrate superior antitumor activity compared to CSPG4 CAR T cells in an orthotopic xenograft murine model of ATC

**Fig. 5:**
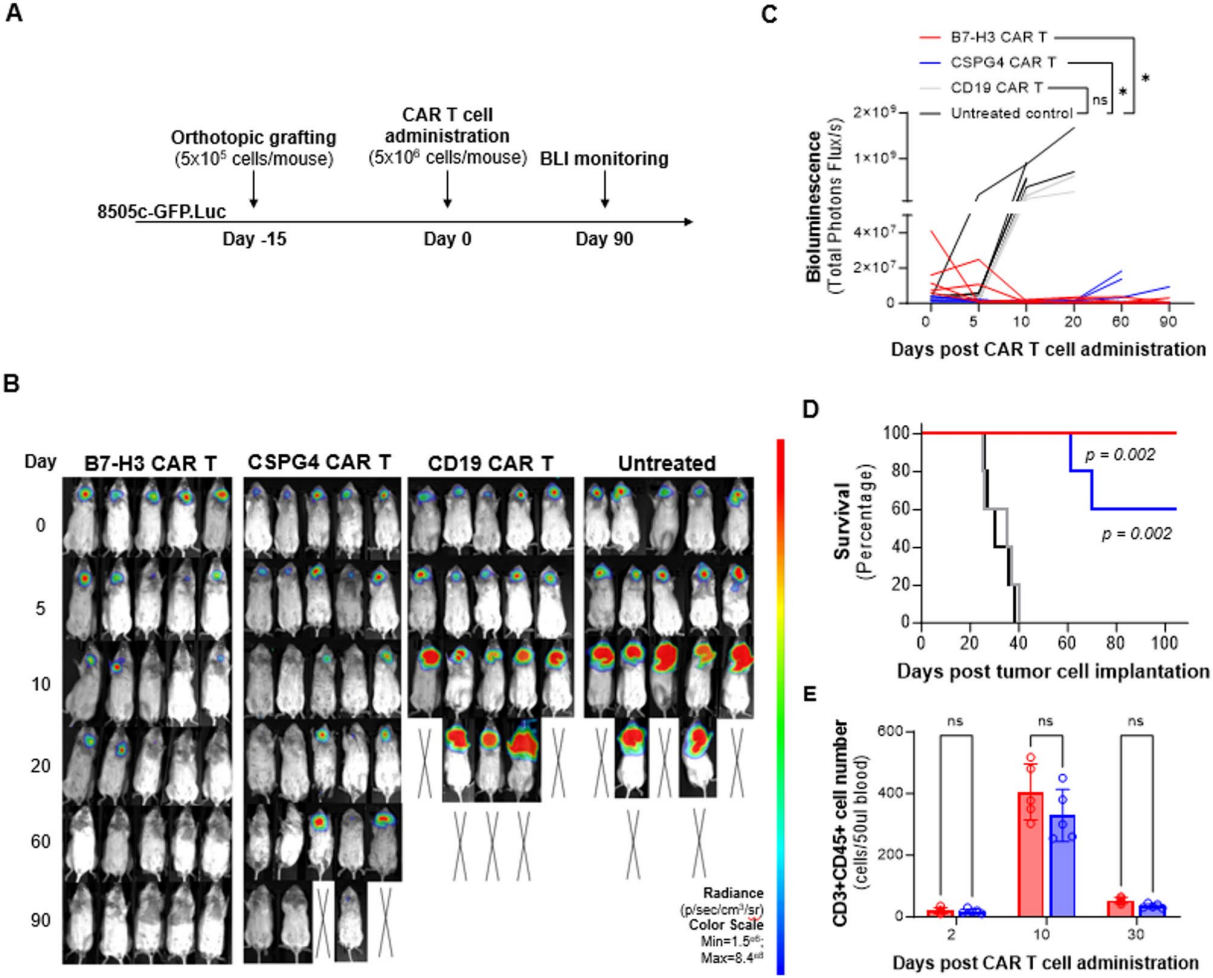
B7-H3 CAR T cells demonstrate superior antitumor activity compared to CSPG4 CAR T cells in an orthotopic murine model of ATC. **(A)** Schematic timeline of the in vivo experiment. **(B)** BLI monitoring of tumor growth in each group treated as indicated (*n* = 5 mice/group). ‘X’ denotes mice dead of disease. **(C)** Total photon flux as a function of time after CAR T cell administration. **(D)** Kaplan-Meier survival curves of mice in each treatment group. **(E)** CAR T cell (CD3 + CD45+) expansion in peripheral blood (50µL) collected from tumor-bearing mice. Statistical analysis was performed using one-way ANOVA multiple comparison test **(C**,** E)**, and log-rank test **(D)**. ns = not significant, *p-value ≤ 0.05, ****p-value ≤ 0.0001

To investigate and compare the long-term in vivo antitumor activity of B7-H3 CAR T cells and CSPG4 CAR T cells, the 8505c-GFP.Luc cell line was orthotopically grafted in NSG mice (Fig. 5A). When the presence of tumors was confirmed by BLI, mice were randomly assigned to the different treatment groups, and CAR T cells were administered as a single dose by tail vein injection (Fig. 5B). B7-H3 CAR T cell administration achieved complete tumor eradication in all treated mice (5/5), which remained tumor-free up to 90 days after treatment. On the other hand, CSPG4 CAR T cells mediated complete tumor eradication only in 2/5 mice and partially controlled tumor growth in 3/5 mice (Fig. 5B, C). B7-H3 CAR T cells and CSPG4 CAR T cells demonstrated similar in vivo proliferation rates, with peak expansion 10 days following administration (Fig. 5E), and they both prolonged the survival of mice when compared to control groups. However, as we observed in our in vitro results, B7-H3 CAR T cell treatment was more effective in eradicating ATC tumors and prolonging mice survival (Fig. 5D), confirming the superiority of B7-H3 as target tumor antigen in ATC.

## Discussion

The design of novel and effective systemic therapies is urgently needed to prolong the survival and improve the quality of life of ATC patients. In this study we provide preclinical evidence supporting CAR T cell-based immunotherapy as a potentially effective treatment for this lethal disease.

Preclinical and clinical evidence supports the rationale to investigate the efficacy of CAR T cell-based immunotherapy with advanced thyroid cancers. Indeed, Min et al. have reported that ICAM-1-redicrected CAR T cells in combination with PD-1 blockade were able to mediate strong antitumor activity against ATC cell lines in vitro and ATC patient-derived xenograft murine models [21, 22]. Furthermore, adoptive transfer of ICAM-1 CAR T cells has been proven safe and feasible in a recent Phase I clinical trial for patients with relapsed/refractory poorly differentiated and anaplastic thyroid cancers [23].

In our previous studies we have demonstrated that the tumor antigens B7-H3 and CSPG4 are highly and homogeneously expressed in different types of solid tumors, with limited distribution on normal tissues [6, 7]. This evidence, along with the pro-tumorigenic functions associated with B7-H3 and CSPG4 expressions, prompted us to develop two second generation CAR constructs designed to specifically recognize these target antigens. We have confirmed the preclinical therapeutic potential of B7-H3-targeted and CSPG4-targeted CAR T cells, by showing that they can induce effective and durable antitumor response in murine models of different types of solid tumors, including pancreatic and ovarian cancer, chordoma, glioblastoma and uveal melanoma [16, 24–26]. The available evidence showing that B7-H3 and CSPG4 are highly expressed in human ATC cell lines and tumors with a limited distribution on adjacent normal thyroid tissue, prompted us to investigate the antitumor efficacy of our CAR T cell therapies with thyroid cancers, including ATC [9, 10, 27]. We confirmed a high and homogeneous gene and protein expression of B7-H3 and CSPG4 in different types of thyroid cancers. Interestingly, we observed that the mRNA level of both TAs was higher in ATC patients as compared to follicular and papillary thyroid cancers, suggesting a potential association between increased B7-H3 and CSPG4 expression levels with a higher degree of tumor aggressiveness. This hypothesis is supported by a previous study showing that increased CSPG4 protein expression was associated with a worse prognosis in ATC patients [10].

In this study we demonstrate that B7-H3-specific and CSPG4-specific CAR T cells are both valuable therapeutic candidates to treat ATC tumors. Our findings indicate that B7-H3 CAR T cells are more effective in eradicating ATC cells in vitro and ATC tumors in mice as compared to CSPG4 CAR T cells. One potential explanation could be related to the higher expression level of B7-H3 on thyroid cancer cells as compared to CSPG4, which might lead to a stronger CAR T cell activation. The hypothesis that CAR T cell-mediated killing correlates with the level of antigen expression is supported by the positive correlation observed between the expression levels of CSPG4 and B7-H3 on thyroid cancer cells and CAR T cell-mediated killing in vitro (Supplementary Fig. 1C, D). This observation might indicate that a higher tumor antigen density on target cells can lead to a stronger CAR T cell activation and enhanced cancer cell killing. The different antitumor activity might also be related to a variance in the binding affinity of the mAbs from which the two CAR constructs have been generated. The B7-H3-specific CAR was generated from the 376.96 mAb, characterized by a moderate binding affinity for the 2Ig isoform of B7-H3 (1.87 × 10^-7^ M) [16]. On the other hand, the binding affinity of the CSPG4-specific 763.74 mAb has been found to be around 1 × 10^-8^ M [28]. Based on this observation, we can conclude that the affinity of the B7-H3 CAR might be lower than the CSPG4 CAR. Interestingly, it has been shown that CAR T cells with lower-affinity scFvs can maintain potent antitumor activity while potentially reducing off-tumor toxicity. As an example, CAR T cells with low affinity scFvs targeting HER2 and CD19 have demonstrated sustained functionality and reduced exhaustion in preclinical murin models and in leukemia patients, respectively [29, 30]. Even though the evidence present in the literature might justify the enhanced efficacy of B7-H3 CAR T cells, further in-depth mechanistic studies are required to better understand the reasons underlying the observed differences in the extent of killing.

As future directions, our study provides the basis to develop combinatorial therapies to enhance CAR T cell antitumor potential by increasing the expression of the target antigen, as well as to design dual-targeting CAR T cell therapy against CSPG4 and B7-H3. Simultaneous targeting of two tumor antigens represents an effective strategy to limit the development of resistance and the risk of relapse associated with tumor antigen escape. Most importantly, the identification of two suitable target antigens strongly supports the design of CAR constructs based on the “AND” logic gating. In this instance CAR T cell activation occurs only when the two antigens are co-expressed by tumor cells. Therefore, such strategy can augment CAR T cell specificity and safety, by reducing the risk of “on-target off-tumor”- associated toxicities. Limitations of this study include the lack of an immunocompetent murine model to reflect the challenges associated with the hostile tumor microenvironment which hinder CAR T cell effector function [31, 32]. Overall, our findings strongly support further investigation for the clinical translation of B7-H3- and CSPG4-targeted CAR T cell-based immunotherapies for the treatment of anaplastic thyroid cancer. Specifically, both tumor antigens should be considered when designing future clinical trials for ATC patients with BRAF wild-type tumors or patients with advanced thyroid cancers relapsed/refractory to first line target therapies.

## Conclusion

Overall, in this study we identified CSPG4 and B7-H3 as valuable therapeutic targets in different types of thyroid carcinoma, including anaplastic thyroid cancer, a rare and lethal disease with no effective treatment. By utilizing preclinical in vitro and in vivo models we demonstrated that CAR T cell-based immunotherapy targeting either tumor antigens represent a valuable alternative treatment option for ATC patients. Our findings provide the translational basis for further exploring CAR T cell immunotherapies targeting CSPG4 and B7-H3 with ATC patients, especially for those who do not respond or relapse after first line treatments.

## Electronic supplementary material

Below is the link to the electronic supplementary material.

Supplementary Material 1

## Data Availability

No datasets were generated or analysed during the current study.

## Abbreviations

ATC: Anaplastic thyroid cancer
B7-H3: B7 homolog 3
BLI: Bioluminescence imaging
CAR: Chimeric antigen receptor
CSPG4: Chondroitin sulphate proteoglycan 4
FDA: Food and Drug Administration
FTC: Follicular thyroid cancer
GFP: Green fluorescent protein
HCC: Hurthle cell carcinoma
mAb: Monoclonal antibody
NSG: NOD.Cg-Prkdcscid Il2rgtm1Wjl/SzJ
PDAC: Pancreatic ductal adenocarcinoma
PTC: Papillary thyroid cancer

## References

[1] Haugen BR, Alexander EK, Bible KC, Doherty GM, Mandel SJ, Nikiforov YE, et al. 2015 American thyroid association management guidelines for adult patients with thyroid nodules and differentiated thyroid cancer: the American thyroid association guidelines task force on thyroid nodules and differentiated thyroid Cancer. Thyroid. 2016;26(1):1–133. 26462967 10.1089/thy.2015.0020 PMC4739132

[2] Smallridge RC, Ain KB, Asa SL, Bible KC, Brierley JD, Burman KD, et al. American thyroid association guidelines for management of patients with anaplastic thyroid cancer. Thyroid. 2012;22(11):1104–39. 23130564 10.1089/thy.2012.0302

[3] Molinaro E, Romei C, Biagini A, Sabini E, Agate L, Mazzeo S, et al. Anaplastic thyroid carcinoma: from clinicopathology to genetics and advanced therapies. Nat Rev Endocrinol. 2017;13(11):644–60. 28707679 10.1038/nrendo.2017.76

[4] Subbiah V, Kreitman RJ, Wainberg ZA, Cho JY, Schellens JHM, Soria JC, Wen PY, Zielinski CC, Cabanillas ME, Boran A, Ilankumaran P, Burgess P, Romero Salas T, Keam B. Dabrafenib plus trametinib in patients with BRAF V600E-mutant anaplastic thyroid cancer: updated analysis from the phase II ROAR basket study. Ann Oncol. 2022 Apr;33(4):406–415. 10.1016/j.annonc.2021.12.014 PMC9338780 35026411

[5] Sterner RC, Sterner RM. CAR-T cell therapy: current limitations and potential strategies. Blood Cancer J. 2021;11(4):69. 33824268 10.1038/s41408-021-00459-7 PMC8024391

[6] Kontos F, Michelakos T, Kurokawa T, Sadagopan A, Schwab JH, Ferrone CR, et al. B7-H3: an attractive target for antibody-based immunotherapy. Clin Cancer Res. 2021;27(5):1227–35. 33051306 10.1158/1078-0432.CCR-20-2584 PMC7925343

[7] Rolih V, Barutello G, Iussich S, De Maria R, Quaglino E, Buracco P, et al. CSPG4: A prototype oncoantigen for translational immunotherapy studies. Journal of Translational Medicine. Volume 15. BioMed Central Ltd.; 2017. 10.1186/s12967-017-1250-4 PMC5494135 28668095

[8] Wang X, Wang Y, Yu L, Sakakura K, Visus C, Schwab JH, et al. CSPG4 in cancer: multiple roles. Curr Mol Med. 2010;10(4):419–29. 20455858 10.2174/156652410791316977

[9] Toda S, Sato S, Saito N, Sekihara K, Matsui A, Murayama D, Nakayama H, Suganuma N, Okubo Y, Hayashi H, Iwasaki H, Miyagi Y, Hoshino D. TROP-2, Nectin-4, GPNMB, and B7-H3 Are Potentially Therapeutic Targets for Anaplastic Thyroid Carcinoma. Cancers (Basel). 2022 Jan 24;14(3):579. 10.3390/cancers14030579 PMC8833363 35158847

[10] Egan CE, Stefanova D, Ahmed A, Raja VJ, Thiesmeyer JW, Chen KJ, et al. CSPG4 is a potential therapeutic target in anaplastic thyroid Cancer. Thyroid. 2021;31(10):1481–93. 34078123 10.1089/thy.2021.0067 PMC8917884

[11] Ilieva KM, Cheung A, Mele S, Chiaruttini G, Crescioli S, Griffin M, et al. Chondroitin sulfate proteoglycan 4 and its potential as an antibody immunotherapy target across different tumor types. Frontiers in Immunology. Volume 8. Frontiers Media S.A.; 2018. 10.3389/fimmu.2017.01911 PMC5767725 29375561

[12] Ventin M, Cattaneo G, Maggs L, Jia J, Arya S, Ferrone S, Wang X, Ferrone CR. B7-H3-targeted CAR T cell activity is enhanced by radiotherapy in solid cancers. Front Oncol. 2023 Jul 7;13:1193963. 10.3389/fonc.2023.1193963 PMC10361748 37483496

[13] Wang Y, Drum DL, Sun R, Zhang Y, Chen F, Sun F, et al. Stressed target cancer cells drive nongenetic reprogramming of CAR T cells and solid tumor microenvironment. Nat Commun. 2023;14(1):5727. 37714830 10.1038/s41467-023-41282-x PMC10504259

[14] Geldres C, Savoldo B, Hoyos V, Caruana I, Zhang M, Yvon E, et al. T lymphocytes redirected against the chondroitin sulfate proteoglycan-4 control the growth of multiple solid tumors both in vitro and in vivo. Clin Cancer Res. 2014;20(4):962–71. 24334762 10.1158/1078-0432.CCR-13-2218 PMC3944408

[15] Ligorio M, Sil S, Malagon-Lopez J, Nieman LT, Misale S, Di Pilato M, et al. Stromal microenvironment shapes the intratumoral architecture of pancreatic Cancer. Cell. 2019;178(1):160–e17527. 31155233 10.1016/j.cell.2019.05.012 PMC6697165

[16] Du H, Hirabayashi K, Ahn S, Kren NP, Montgomery SA, Wang X, et al. Antitumor responses in the absence of toxicity in solid tumors by targeting B7-H3 via chimeric antigen receptor T cells. Cancer Cell. 2019;35(2):221–e2378. 30753824 10.1016/j.ccell.2019.01.002 PMC6645919

[17] Kageshita Toshiro HSOTFS. Human high molecular weight-melanoma associated antigen mimicry by mouse anti-idiotypic MAb MK2-23. Immunohistochemical analysis of the reactivity of anti-anti-idiotypic MAb with surgically removed melanoma lesions. Int J Cancer. 1995;60(3):334–40. 7829240 10.1002/ijc.2910600310

[18] Wang X, Osada T, Wang Y, Yu L, Sakakura K, Katayama A, et al. CSPG4 protein as a new target for the antibody-based immunotherapy of triple-negative breast cancer. J Natl Cancer Inst. 2010;102(19):1496–512. 20852124 10.1093/jnci/djq343 PMC2950168

[19] Nucera C, Nehs MA, Mekel M, Zhang X, Hodin R, Lawler J, et al. A novel orthotopic mouse model of human anaplastic thyroid carcinoma. Thyroid. 2009;19(10):1077–84. 19772429 10.1089/thy.2009.0055 PMC2833178

[20] Haase J, Misiak D, Bauer M, Pazaitis N, Braun J, Pötschke R, et al. IGF2BP1 is the first positive marker for anaplastic thyroid carcinoma diagnosis. Mod Pathol. 2021;34(1):32–41. 32719445 10.1038/s41379-020-0630-0 PMC7806508

[21] Min IM, Shevlin E, Vedvyas Y, Zaman M, Wyrwas B, Scognamiglio T, et al. CAR T therapy targeting ICAM-1 eliminates advanced human thyroid tumors. Clin Cancer Res. 2017;23(24):7569–83. 29025766 10.1158/1078-0432.CCR-17-2008 PMC5732861

[22] Gray KD, McCloskey JE, Vedvyas Y, Kalloo OR, Eshaky S, El, Yang Y, et al. PD1 Blockade enhances ICAM1-Directed CAR T therapeutic efficacy in advanced thyroid Cancer. Clin Cancer Res. 2020;26(22):6003–16. 32887724 10.1158/1078-0432.CCR-20-1523 PMC7709864

[23] Hsu JM, Von Hofe E, Hsu YM, Shieh JH, Chaekal OK, Guarneri D, et al. Phase I study of AIC100 in relapsed and/or refractory advanced thyroid cancer and anaplastic thyroid cancer. J Clin Oncol. 2022;40:16.

[24] Wang K, Osei-Hwedieh DO, Walhart TA, Hung YP, Wang Y, Cattaneo G, Ma T, Dotti G, Wang X, Ferrone S, Schwab JH. B7-H3 CAR-T cell therapy combined with irradiation is effective in targeting bulk and radiation-resistant chordoma cancer cells. J Immunother Cancer. 2025 Jan 22;13(1):e009544. 10.1136/jitc-2024-009544 PMC11784168 39848690

[25] Pellegatta S, Savoldo B, Ianni N, Di, Corbetta C, Chen Y, Patané M et al. Constitutive and TNFα-inducible expression of chondroitin sulfate proteoglycan 4 in glioblastoma and neurospheres: Implications for CAR-T cell therapy [Internet]. Vol. 10, Sci. Transl. Med. 2018. Available from: https://www.science.org 10.1126/scitranslmed.aao2731 PMC8713441 29491184

[26] Ventin M, Cattaneo G, Arya S, Jia J, Gelmi MC, Sun Y, et al. Chimeric antigen receptor T cell with an inducible Caspase-9 suicide gene eradicates uveal melanoma liver metastases via B7-H3 targeting. Clin Cancer Res. 2024;30(15):3243–58. 38767611 10.1158/1078-0432.CCR-24-0071 PMC11572477

[27] Song P, Xu Y, Ye G. B7-H3 and ICAM-1 are potentially therapeutic targets for thyroid carcinoma. Diagn Pathol. 2024 Jun 10;19(1):77. 10.1186/s13000-024-01504-2 PMC11163747 38858715

[28] Giacomini PNPFS. Analysis of the interaction between a human high molecular weight melanoma-associated antigen and the monoclonal antibodies to three distinct antigenic determinants. J Immunol. 1985;135(1):696–702. 2582052

[29] Ghorashian S, Kramer AM, Onuoha S, Wright G, Bartram J, Richardson R, et al. Enhanced CAR T cell expansion and prolonged persistence in pediatric patients with ALL treated with a low-affinity CD19 CAR. Nat Med. 2019;25(9):1408–14. 31477906 10.1038/s41591-019-0549-5

[30] Liu X, Jiang S, Fang C, Yang S, Olalere D, Pequignot EC, et al. Affinity-tuned ErbB2 or EGFR chimeric antigen receptor T cells exhibit an increased therapeutic index against tumors in mice. Cancer Res. 2015;75(17):3596–607. 26330166 10.1158/0008-5472.CAN-15-0159 PMC4560113

[31] Xu Z, Shin HS, Kim YH, Ha SY, Won JK, Kim SJ, et al. Modeling the tumor microenvironment of anaplastic thyroid cancer: an orthotopic tumor model in C57BL/6 mice. Front Immunol. 2023;14:1187388. 37545523 10.3389/fimmu.2023.1187388 PMC10403231

[32] Ventin M, Cattaneo G, Maggs L, Arya S, Wang X, Ferrone CR. Implications of high tumor burden on chimeric antigen receptor T-Cell immunotherapy: A review. JAMA Oncol. 2024;10(1):115–21. 37943567 10.1001/jamaoncol.2023.4504

[33] Schanda N, Sauer T, Kunz A, Hückelhoven-Krauss A, Neuber B, Wang L, Hinkelbein M, Sedloev D, He B, Schubert ML, Müller-Tidow C, Schmitt M, Schmitt A. Sensitivity and specificity of CD19.CAR-T cell detection by flow cytometry and PCR. Cells. 2021;10(11):3208. 34831430 10.3390/cells10113208 PMC8621201

