# Supplementary material for: B7-H3 and CSPG4-targeted CAR T cells as potent effectors in anaplastic thyroid cancer
Source: J Exp Clin Cancer Res. 2025 Aug 22;44:248. doi: 10.1186/s13046-025-03475-8 (PMC12372207; doi:10.1186/s13046-025-03475-8)
Supplement: Supplementary file 1 — Supplementary Material 1 [file 13046_2025_3475_MOESM1_ESM.docx]

**Supplementary Table 1. Thyroid cancer patients’ clinical information and score of CSPG4 expression.**

| **Age** | **Sex** | **Pathology diagnosis** | **TNM** | **Stage** | **Positively stained cells (%) *** | **Intensity**** | **Score***** |
| --- | --- | --- | --- | --- | --- | --- | --- |
| 25 | F | PTC | T3N0M0 | I | 3 | 2 | 5 |
| 28 | F | PTC | T3N0M0 | I | 1 | 1 | 2 |
| 52 | F | PTC | T2N0M0 | I | 1 | 1 | 2 |
| 35 | F | PTC | T3N1M0 | I | 2 | 1 | 3 |
| 24 | M | PTC | TN0M0 | I | 3 | 2 | 5 |
| 37 | F | PTC | T4N1M0 | I | 3 | 2 | 5 |
| 31 | F | PTC | T3N0M0 | I | 3 | 2 | 5 |
| 26 | M | PTC | T4N1M0 | I | 3 | 2 | 5 |
| 19 | F | PTC | T2N1M0 | I | 3 | 2 | 5 |
| 36 | F | PTC | T2N0M0 | I | 3 | 1 | 4 |
| 46 | F | PTC | T2N0M0 | I | 3 | 1 | 4 |
| 40 | F | PTC | T2N0M0 | I | 3 | 1 | 4 |
| 34 | F | PTC | T1N0M0 | I | 3 | 1 | 4 |
| 53 | F | PTC | T3N0M0 | I | 3 | 2 | 5 |
| 42 | F | PTC | T1N0M0 | I | 3 | 1 | 4 |
| 35 | F | PTC | T1bN0M0 | I | 3 | 2 | 5 |
| 52 | F | PTC | T2N0M0 | I | 3 | 1 | 4 |
| 39 | F | PTC | T2N0M0 | I | 3 | 1 | 4 |
| 48 | F | PTC | T2N0M0 | I | 1 | 1 | 2 |
| 46 | M | PTC | T3N0M0 | I | 3 | 2 | 5 |
| 50 | M | PTC | T3N0M0 | I | 3 | 1 | 4 |
| 48 | F | PTC | T2N0M0 | I | 3 | 2 | 5 |
| 24 | F | PTC | T3N0M0 | I | 3 | 1 | 4 |
| 24 | F | PTC | T2N0M0 | I | 3 | 2 | 5 |
| 68 | M | PTC | T2N0M0 | I | 3 | 1 | 4 |
| 44 | M | FTC | T4N0M0 | I | 3 | 2 | 5 |
| 70 | F | FTC | T3N1M0 | II | 3 | 2 | 5 |
| 72 | F | FTC | T3N0M0 | II | 3 | 1 | 4 |
| 52 | F | FTC | T3N0M0 | I | 3 | 2 | 5 |
| 41 | M | FTC | T4N1M0 | I | 3 | 2 | 5 |
| 53 | M | FTC | T3N0M0 | I | 3 | 1 | 4 |
| 41 | F | FTC | T2N0M0 | I | 3 | 1 | 4 |
| 57 | F | FTC | T4aN0M0 | III | 3 | 2 | 5 |
| 68 | F | FTC | T4aN0M0 | III | 3 | 1 | 4 |
| 66 | F | FTC | T3N0M0 | II | 3 | 1 | 4 |
| 63 | F | FTC | T4aN0M0 | III | 3 | 1 | 4 |
| 55 | F | FTC | T4aN0M0 | III | 3 | 2 | 5 |
| 56 | M | FTC | T3N0M0 | II | 3 | 1 | 4 |
| 27 | M | FTC | T2N0M0 | I | 3 | 1 | 4 |
| 63 | F | FTC | T4N0M1 | IVB | 3 | 1 | 4 |
| 36 | F | FTC | T3N0M0 | I | 3 | 1 | 4 |
| 29 | F | FTC | T1N0M0 | I | 3 | 1 | 4 |
| 49 | F | FTC | T2N0M0 | I | 3 | 1 | 4 |
| 66 | M | FTC | T3aN0M0 | II | 3 | 2 | 5 |
| 48 | F | FTC | T1N0M0 | I | 3 | 1 | 4 |
| 70 | F | FTC | T2N0M0 | I | 3 | 1 | 4 |
| 49 | F | FTC | T2N0M0 | I | 3 | 1 | 4 |
| 30 | F | ATC | T4N0M0 | IVB | 3 | 1 | 4 |
| 37 | M | ATC | T2N0M0 | IVA | 2 | 1 | 3 |
| 54 | M | ATC | T3N0M0 | IVA | 3 | 1 | 4 |
| 86 | F | ATC | T4N0M0 | IVB | 3 | 1 | 4 |
| 78 | F | ATC | T4N0M0 | IVB | 3 | 1 | 4 |
| 42 | M | ATC | T4N0M0 | IVB | 2 | 1 | 3 |
| 47 | M | ATC | T2N0M0 | IVA | 3 | 1 | 4 |
| 69 | M | ATC | T4N0M0 | IVB | 2 | 1 | 3 |
| 64 | F | ATC | T2N0M0 | IVA | 3 | 1 | 4 |
| 39 | F | ATC | T4N0M0 | IVB | 3 | 1 | 4 |
| 49 | F | ATC | T4N0M0 | IVB | 3 | 2 | 5 |
| 57 | M | ATC | T4N0M0 | IVB | 3 | 1 | 4 |
| 38 | M | Normal thyroid | - | - | 1 | 1 | 2 |
| 30 | M | Normal thyroid | - | - | 1 | 1 | 2 |
| 28 | M | Normal thyroid | - | - | 1 | 1 | 2 |
| 19 | M | Normal thyroid | - | - | 1 | 1 | 2 |
| 21 | F | Normal thyroid | - | - | 1 | 1 | 2 |
| 40 | M | Normal thyroid | - | - | 1 | 1 | 2 |
| 50 | M | Normal thyroid | - | - | 1 | 1 | 2 |
| 45 | M | Normal thyroid | - | - | 0 | 0 | 0 |
| 15 | F | Normal thyroid | - | - | 1 | 1 | 2 |

PTC = papillary thyroid carcinoma, FTC = follicular thyroid carcinoma, ATC = anaplastic thyroid cancer. TNM and clinical stage (AJCC version 8).

*Percentage of core stained (0 = negative, 1 = 1–29%, 2 = 30–59%, 3 = 60–100%); **intensity of staining (0 = negative, 1 = weak, 2 = intermediate, 3 = strong); ***score graded as negative (0), weak (1–2), intermediate (3–4) and strong (5).

p= 0.621

p= 0.339

**C**

**D**


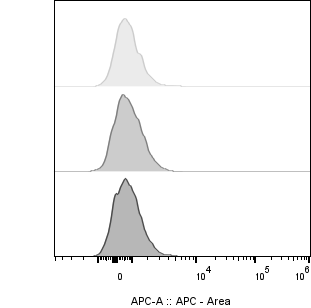


Isotype

B7-H3

CSPG4


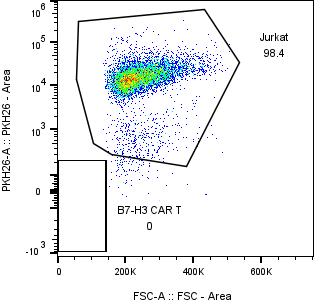

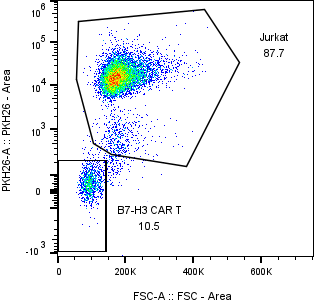

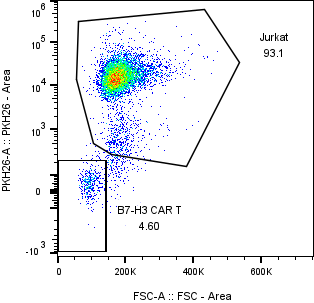


Jurkat

(untreated control)

Jurkat + B7-H3 CAR T

(E:T = 1:1)

Jurkat + B7-H3 CAR T

(E:T = 1:2)


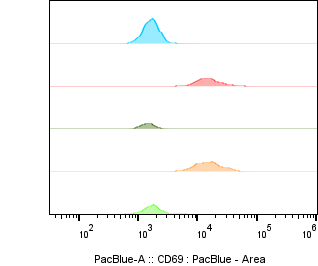


Non stimulated CAR T

B7-H3 CAR T+8505c

CSPG4 CAR T+8505c

CSPG4 CAR T+Jurkat

B7-H3 CAR T+Jurkat


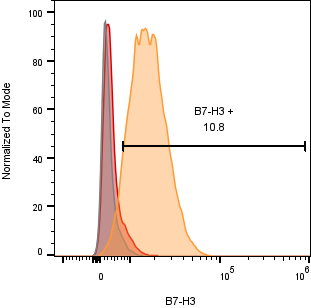


Isotype

B7-H3^WT^

B7-H3^-/-^

MDA-MB-231


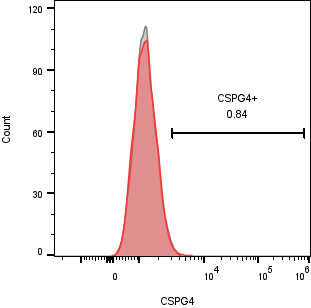


Isotype

CSPG4

PDAC6

Jurkat

**E**

**F**

**G**

**H**

**I**

**A**

**B**


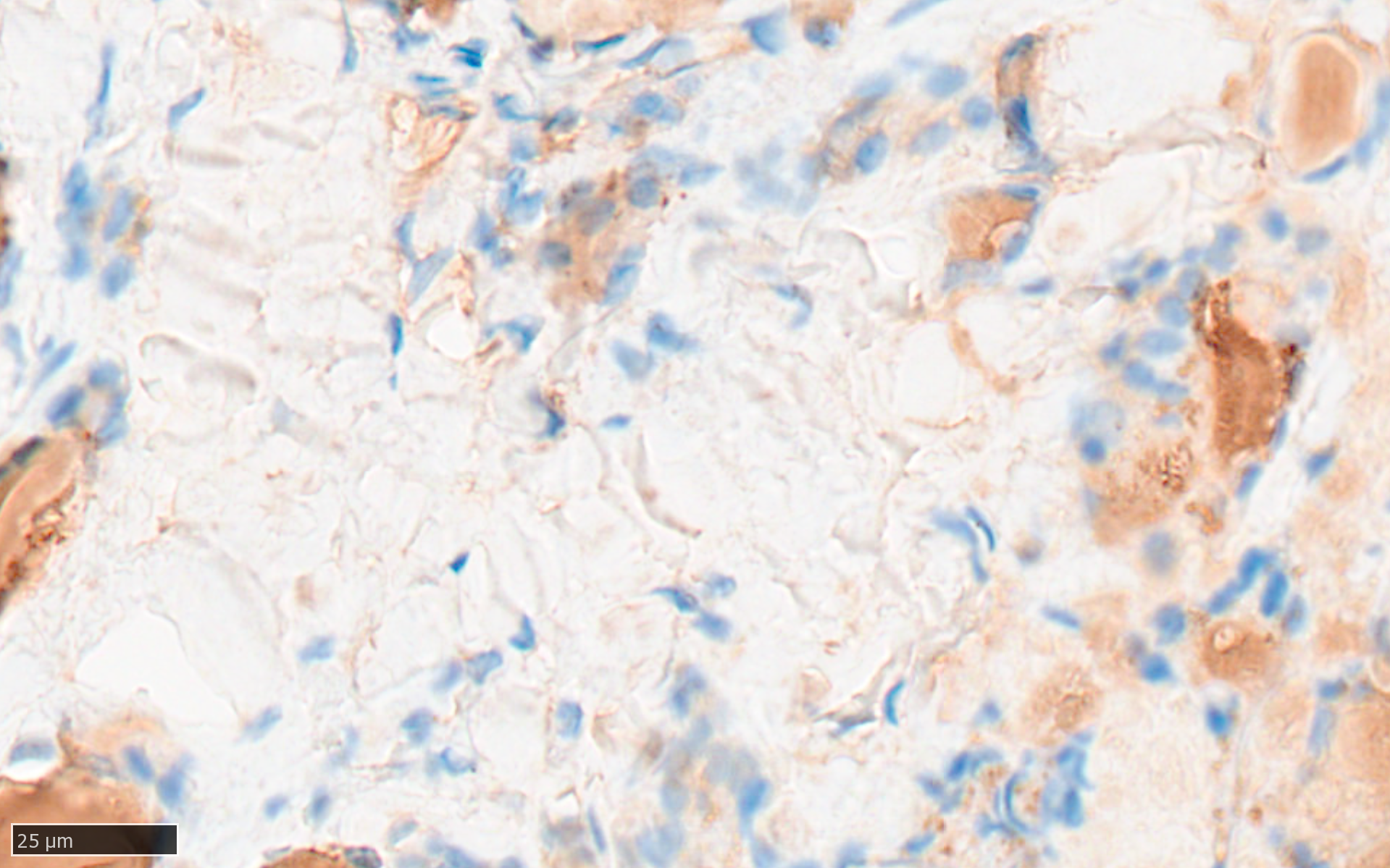


Negative expression

(normal thyroid tissue)


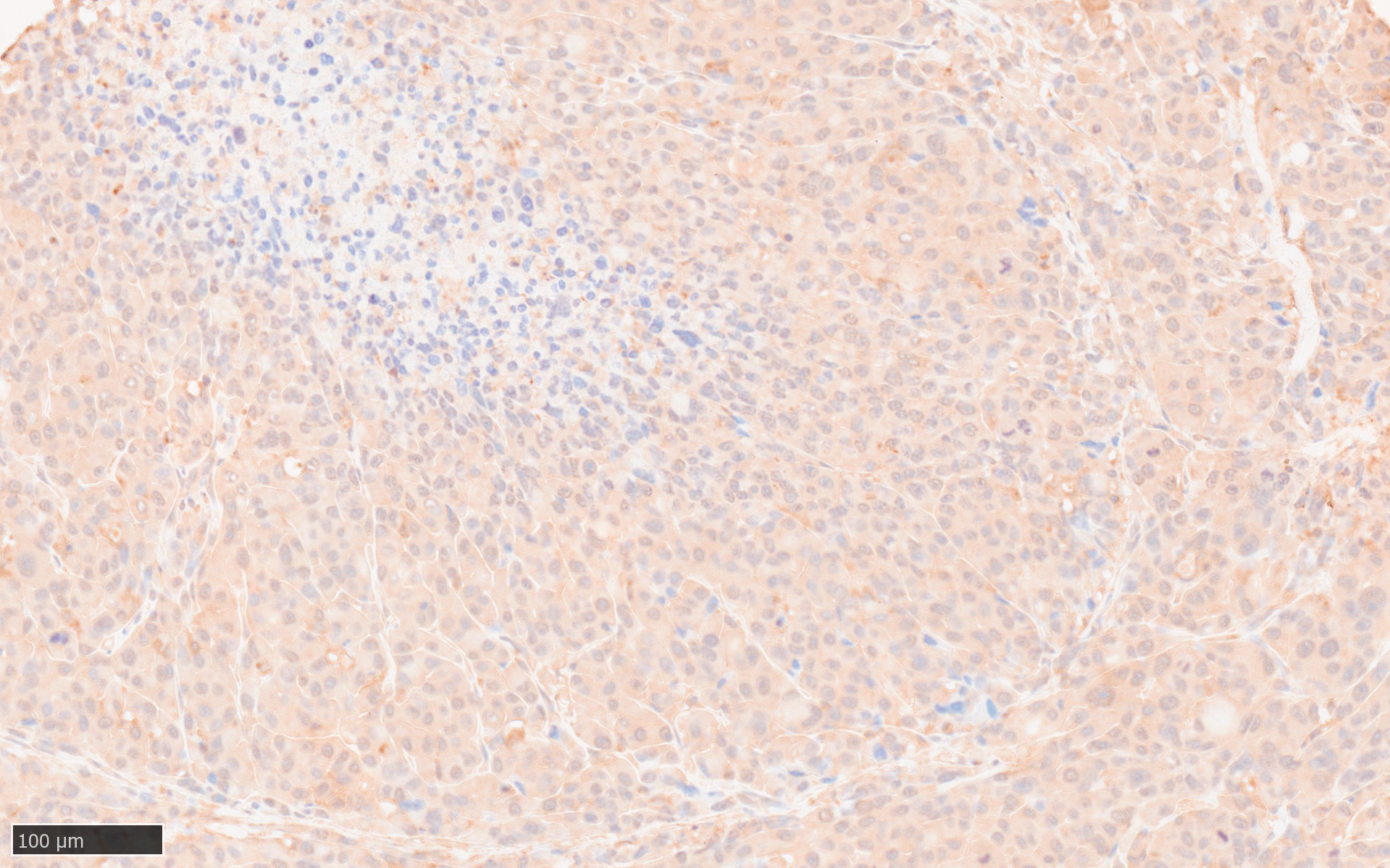

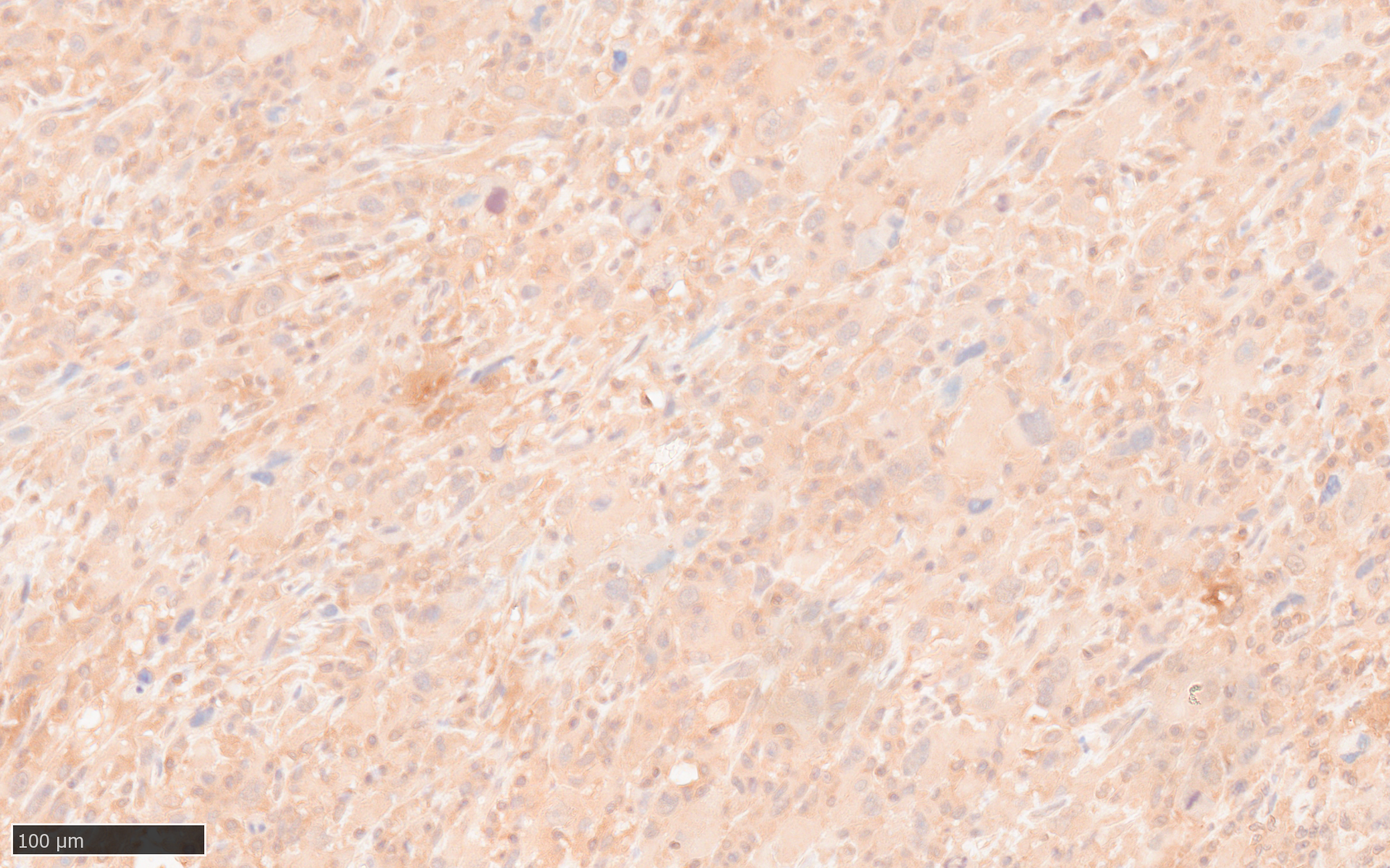


Percentage score = 2 (30-29%)

Percentage score = 3 (60-100%)



**Supplementary Fig. 1. Positive correlation between target antigen expression and CAR T cell-mediated killing and lack of ‘off-tumor’ toxicity induced by CAR T cell treatment *in vitro*. A)** Representative image showing negative CSPG4 expression on normal thyroid tissue assessed by IHC staining with the CSPG4-specific 763.74 mAb. **B)** Representative images showing different scores of CSPG4 expression on thyroid cancers determined by measuring the % of positive cells in each tissue core. **C, D)** The mean fluorescence intensity (MFI) of CSPG4 **(C)** and B7-H3 **(D)** expressions on thyroid cancer cell lines is represented on the x axis, while the y axis displays the percentage of cancer cell killing mediated by CAR T cells. Each dot represents a thyroid cancer cell line. **E)** CSPG4 and B7-H3 expression on Jurkat cells and PDAC6 cells was assessed by flow cytometry by utilizing the CSPG4-specific 763.74 mAb and the B7-H3-specific 367.96 mAb, respectively. **F, G)** The number of residual Jurkat cells following coculture with both CAR T cell types was assessed by flow cytometry. Representative dot plots are showed. **H)** CD69 expression on CAR T cells was assessed by flow cytometry following a 24-hour coculture period with target cells. **I)** B7-H3 expression on MDA-MB-231 B7-H3-/- cells was assessed by flow cytometry.

**
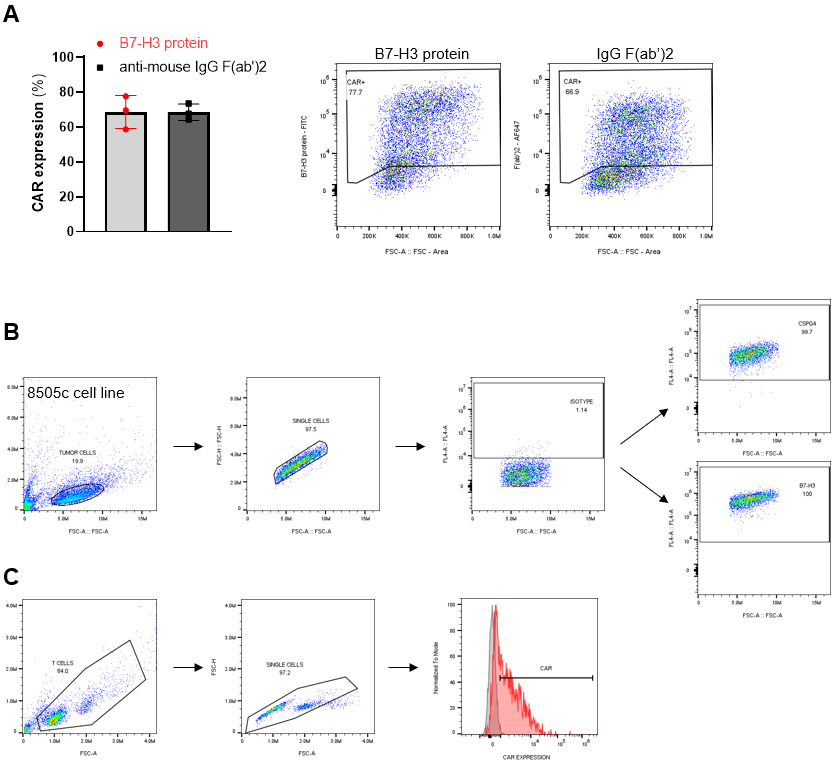
**

**Supplementary Fig. 2. Gating strategies utilized to perform flow cytometric analyses. A)** B7-H3 CAR expression on transduced T cells was assessed by flow cytometry by utilizing two different detection methods, specifically a FITC-labeled recombinant B7-H3 protein and the anti-mouse IgG F(ab’)2. **B)** Representative dot plots showing the gating strategy utilized to determine CSPG4 and B7-H3 expression on the 8505c cell line. The described gating strategy was applied to all the analyzed thyroid cancer cell lines. **C)** Representative dot plots showing the gating strategy utilized to determine CAR expression on transduced T cells. The described gating scheme was applied to both B7-H3 CAR T cells and CSPG4 CAR T cells.
